# Oxygen Vacancies and Surface Wettability: Key Factors in Activating and Enhancing the Solar Photocatalytic Activity of ZnO Tetrapods

**DOI:** 10.3390/ijms242216338

**Published:** 2023-11-15

**Authors:** Farid Orudzhev, Arsen Muslimov, Daud Selimov, Rashid R. Gulakhmedov, Alexander Lavrikov, Vladimir Kanevsky, Rashid Gasimov, Valeriya Krasnova, Dinara Sobola

**Affiliations:** 1 Smart Materials Laboratory, Dagestan State University, 367000 Makhachkala, Russia; daud-selimov@live.com (D.S.); raxa.keyt@gmail.com (R.R.G.); 2 Federal Research Center “Crystallography and Photonics”, Russian Academy of Sciences, 119333 Moscow, Russia; amuslimov@mail.ru (A.M.); a.lawrikow@mail.ru (A.L.); kanev@crys.ras.ru (V.K.); valeriya070707@inbox.ru (V.K.); 3 Institute of Radiation Problems of Azerbaijan National Academy of Sciences, AZ1143 Baku, Azerbaijan; 4 Department of Physics, Faculty of Electrical Engineering and Communication, Brno University of Technology, 61600 Brno, Czech Republic

**Keywords:** zinc oxide, wettability, oxygen vacancies, sunlight, photocatalysis

## Abstract

This paper reports on the high photocatalytic activity of ZnO tetrapods (ZnO-Ts) using visible/solar light and hydrodynamic water flow. It was shown that surface oxygen defects are a key factor in the photocatalytic activity of the ZnO-Ts. The ability to control the surface wettability of the ZnO-Ts and the associated concentration of surface defects was demonstrated. It was demonstrated that the photocatalytic activity during the MB decomposition process under direct and simulated sunlight is essentially identical. This presents excellent prospects for utilizing the material in solar photocatalysis.

## 1. Introduction

A global environmental problem is the presence of organic substances as pollutants in industrial wastewater, household waste, and landfills. Over many decades, new alternative methods of wastewater treatment have been developed. One of the most attractive methods in terms of cost-effectiveness, efficiency, and simplicity of technology is photocatalytic water purification [1,2,3]. TiO_2_ and ZnO are widely used in photocatalysis due to their high physicochemical stability, low toxicity, cost-effectiveness, and availability [4]. However, they have some drawbacks, such as a wide bandgap and the fast recombination of photoinduced charges. To improve the photoresponse of zinc oxide and prevent the recombination of e^−^ and h^+^, it undergoes extensive modifications, including metal and non-metal doping, the deposition of noble metals, and the construction of heterojunctions [5]. The popularity of ZnO is explained by its variety of morphological forms with different optical properties and types of defects such as nanoparticles [6], nanorods [7], nanotubes [8], nanosheets [9], and tetrapods [10]. These branched nanostructured materials predominantly high surface areas and good dispersion, which prevents them from forming aggregates and improves their photocatalytic characteristics [7].

One of the methods of increasing photocatalytic activity is the controlled synthesis of materials, allowing for the creation of intrinsic defects in the structural matrix without introducing impurities. Zinc oxide is known for numerous defect states, such as zinc vacancies (V_Zn_), oxygen vacancies (V_O_), interstitial zinc (Zn_i_), oxygen incorporation (O_i_), zinc anti-sites (Zn_O_), and oxygen anti-sites (O_Zn_) [11]. Among them, oxygen vacancies are of particular interest due to their ability to enhance light absorption in the visible range by forming isolated energy sublevels in the bandgap [12,13], suppressing the recombination of photo-generated charges and increasing O_2_ adsorption, which, in turn, enhances the generation of superoxide radicals ·O_2_^−^ [14].

Wettability is an important component of photocatalysis, indicating the physical interaction between a liquid and the surface of a material. It depends on the chemical composition, surface free energy, and geometric structure of the surface [15]. In recent years, one of the simplest and most popular methods for controlling wettability has been UV irradiation, which allows for reversible transitions between superhydrophilicity and superhydrophobicity [16,17,18]. In [19], the reversible wettability of ZnO thin films under light irradiation was investigated. Schematically, wettability switching under these conditions can be described as follows: upon illumination, formed holes react with lattice oxygen to create oxygen vacancies, which can react with water molecules or oxygen to form hydroxyl groups, increasing hydrophilicity [20]. Recent studies indicate that improving control over the wettability of a catalyst’s surface can lead to an increase in its photocatalytic activity [21,22,23]. Often, surface modification using compounds such as hexamethyldisilazane, perfluorodecyltriethoxysilane, 3-(methacryloxy) propyltrimethoxysilane, and trimethylchlorosilane is used to achieve controlled changes in the wettability of photocatalyst surfaces. However, it should be noted that these compounds, by occupying active sites on the surface, may have a negative impact on surface chemical reactions and the interaction of the catalyst with light. Therefore, it is important to develop unmodified photocatalysts with the ability to control surface wettability to achieve optimal catalytic characteristics. In this regard, catalysts with simultaneous control over surface wettability and defect engineering represent a special scientific and practical interest.

This paper presents the results of the carbothermal synthesis of high-defect ZnO microtetrapods which are active in visible light and investigates the influence of surface wettability switching on their photocatalytic activity.

## 2. Results and Discussion

**Figure 1:**
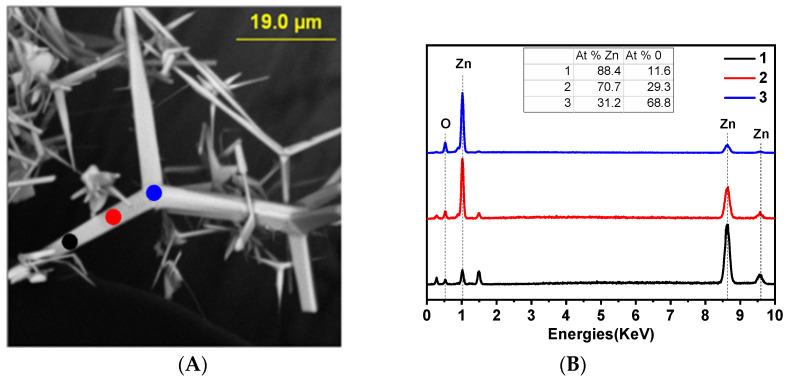

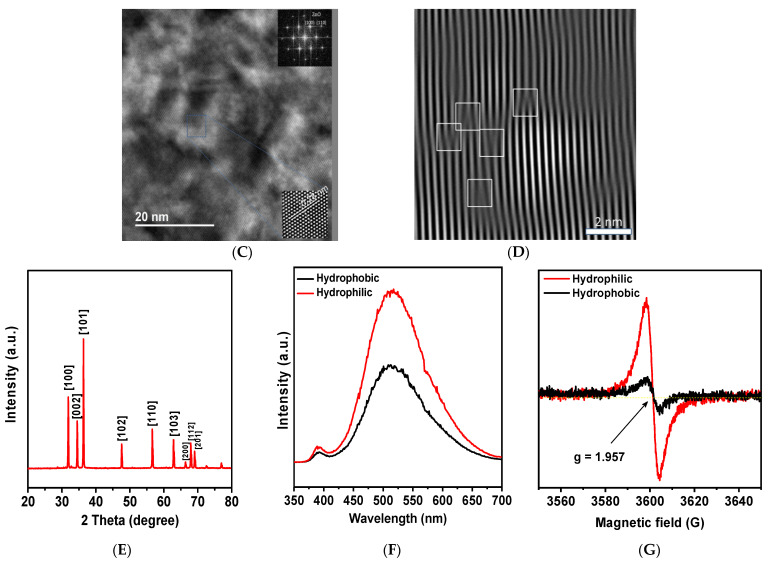
(**A**) SEM image. (**B**) EDX analysis. (**C**) HR-TEM image of a cross-section of the “legs” of a ZnO-T. Inset: magnified image of the highlighted area and its Fourier transform. (**D**) FFT image of the “legs” of a ZnO-T. (**E**) XRD spectra of ZnO-Ts. (**F**) Cathodoluminescence spectra. (**G**) ESR spectra.

During the carbothermal synthesis process, tetrapods (self-organized, pseudo-three-dimensional nanostructures characterized by four monocrystalline rods emanating from the vertices of a tetrahedron) of ZnO were formed with “legs” whose lengths (Figure 1A) were up to tens of micrometers.

The EDS spectra (Figure 1B) show the atomic ratios of a ZnO-T in three positions from the center to the tip. From the obtained results, the ratio of the atomic masses of O and Zn changes. In the center, there is a significant predominance of oxygen, approximately 68.8%, while the content of zinc is about 31.2%. In the central region of the tetrapod, this ratio is inversely proportional: 29.3% O and 70.7% Zn. At the tip, zinc dominates, with its content increasing to 88.4%, and the oxygen content is approximately 11.6%. This indicates that there is a high accumulation of oxygen vacancies (V_O_) at the tips, while at the base of the tetrapod, there is a deficiency of zinc, suggesting the presence of interstitial zinc vacancies (Zni).

According to transmission electron microscopy data (Figure 1C) of a perpendicular cross-section of one of the ZnO-T rods, a monocrystalline structure is formed. An analysis of diffraction patterns and calculations of interplanar distances confirm the wurtzite structure of ZnO-T [JCPDS № 79–0205]. The absence of extended defects demonstrates the high crystalline quality of the ZnO-T. However, an analysis using Fourier transformation and subsequent image filtering (Figure 1D) show the presence of broken planes that are packaging defects and possible dislocation cores. The band structure is distorted around the dislocation core, and an additional level is introduced closer to the center of the forbidden zone. In n-type crystals such as ZnO, dislocations can capture electrons and hinder their recombination, which is also a factor that increases photosensitivity.

**Figure 2:**
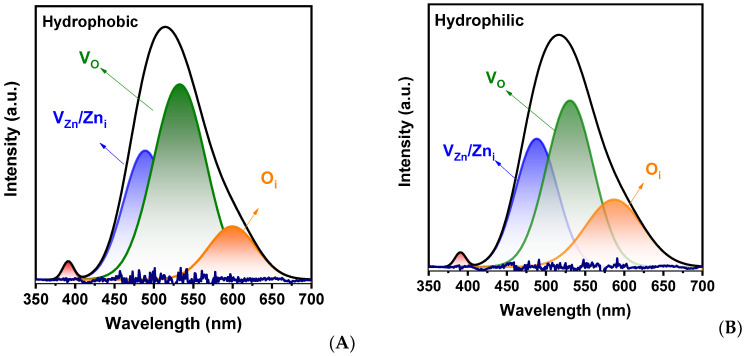
Gauss fitted CL spectra of T-ZnO samples: hydrophobic (**A**) and hydrophilic (**B**).

Diffraction reflections on the X-ray diffraction spectrum of the tetrapods (Figure 1E) corresponded to the hexagonal wurzite phase of ZnO, with a slight shift toward higher angles compared to the reference sample [JCPDS No. 79-0205], indicating a reduction in the size of the ZnO unit cell. The reduction in ZnO parameters is likely associated with point defects such as oxygen and zinc vacancies. The cathodoluminescence spectra in Figure 1F for hydrophilic and hydrophobic zinc oxide show a narrow band in the near UV region, corresponding to excitonic emission, and a broader and more intense band in the visible region associated with intrinsic or impurity point defects such as oxygen vacancies (V_O_), oxygen interstitials (O_s_), zinc vacancies (VZn_s_), and zinc interstitials (Zn_s_) and their complexes. The cathodoluminescence spectra were fitted with Gaussian functions to determine the components. The results are shown in Figure 2. Additionally, from Figure 1F, it can be concluded that the intensity of the defect band increases when transitioning from hydrophobic to hydrophilic states.

Figure 1G shows that the ESR spectrum represents a symmetrical singlet with parameters g = 1.957 and ∆B = 6.11 G, and these parameter values are nearly identical for both samples. The identity of the g-factor values and line width indicates that the same paramagnetic center is formed in all samples in terms of both its chemical nature and the structure of its immediate environment. These paramagnetic centers are attributed to point defects. The fact that the g-factor values are lower than the g-factor value for a free electron (2.0023) suggests that these defects have a hole-like character and carry a positive charge (V-centers). In [11], the signal with g~1.95–1.97 is associated with oxygen vacancies V_O_ specifically in ZnO powders. It is evident that the concentration of defects in the hydrophobic sample is significantly lower compared to the hydrophilic sample, and we can conclude that the concentration of oxygen vacancies has increased. For oxygen vacancies in the neutral V_O_ and 2+ charge states V_O_^2+^, a localized occupied state is recognized in the bandgaps at 2.5–2.6 eV and 0.9–1 eV below the conduction-band minimum [24], respectively, and this suggests the activity of the ZnO tetrapods in the visible region of the spectrum.

**Figure 3:**
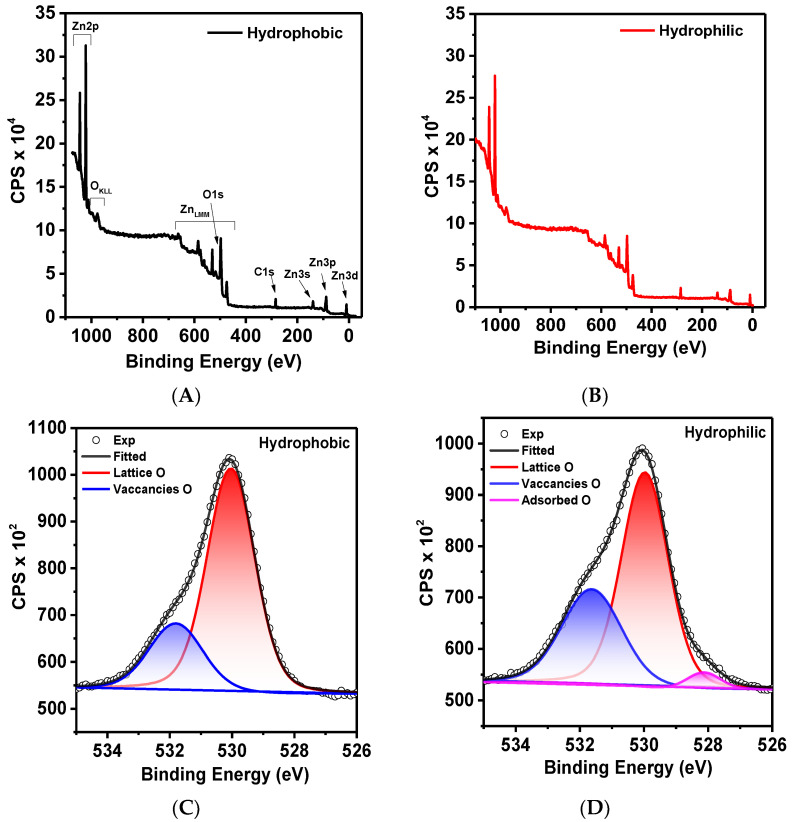

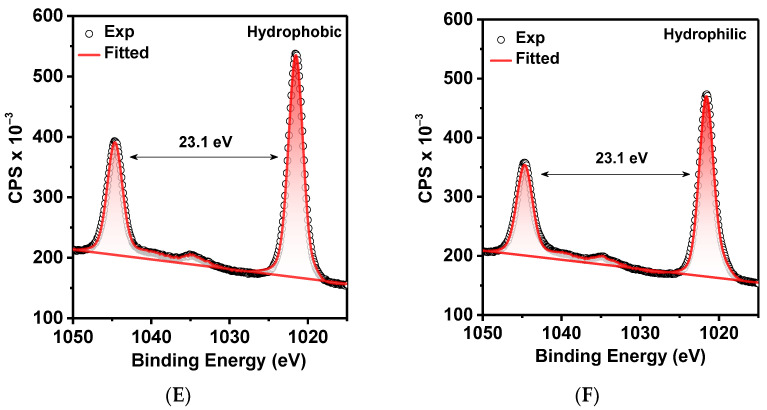
XPS spectra (**A**,**B**) wide XPS (**C**,**D**) O1s spectra and (**E**,**F**) Zn2p spectra of hydrophobic and hydrophilic ZnO-Ts.

The XPS spectra (Figure 3) were calibrated using the C1s peak (284.6 eV). From the survey spectra (Figure 3A,B), it can be concluded that the surface is chemically pure and free from impurities. There are no visible differences between the hydrophilic and hydrophobic samples. In the high-resolution Zn2p spectra (Figure 3E,F), two distinct doublet peaks can be observed at 1021.6 eV and 1044.7 eV, corresponding to Zn2p3/2 and Zn2p1/2, respectively. The energy difference of 23.1 eV falls within the standard reference resolution of ZnO, and the visible doublets are attributed to Zn^2+^ ions. The asymmetric O1s peak is presented in Figure 3C,D. For the hydrophobic state, the spectrum is approximated by two components with maxima at 530.11 eV and 531.28 eV, corresponding to different forms of oxygen. The first peak at 530.11 eV can be attributed to oxygen ions (O^2−^) in the wurtzite structure of the ZnO. The higher energy peak at 531.28 eV is associated with regions of oxygen deficiency or oxygen vacancies in the matrix [25,26]. In the hydrophilic state, as shown in Figure 3D, an additional peak appears at a binding energy of 528 eV. The presence of peaks in this region is usually attributed to adsorbed oxygen [27]. The adsorption of oxygen on the hydrophilic surface can be explained by the surface’s tendency for charge compensation. Additionally, it is interesting to compare the ratio of the integrated peak areas of lattice oxygen and oxygen vacancies. It can be observed that the ratio changes from 3 to 1.8 when transitioning from the hydrophobic to the hydrophilic state.

**Figure 4:**
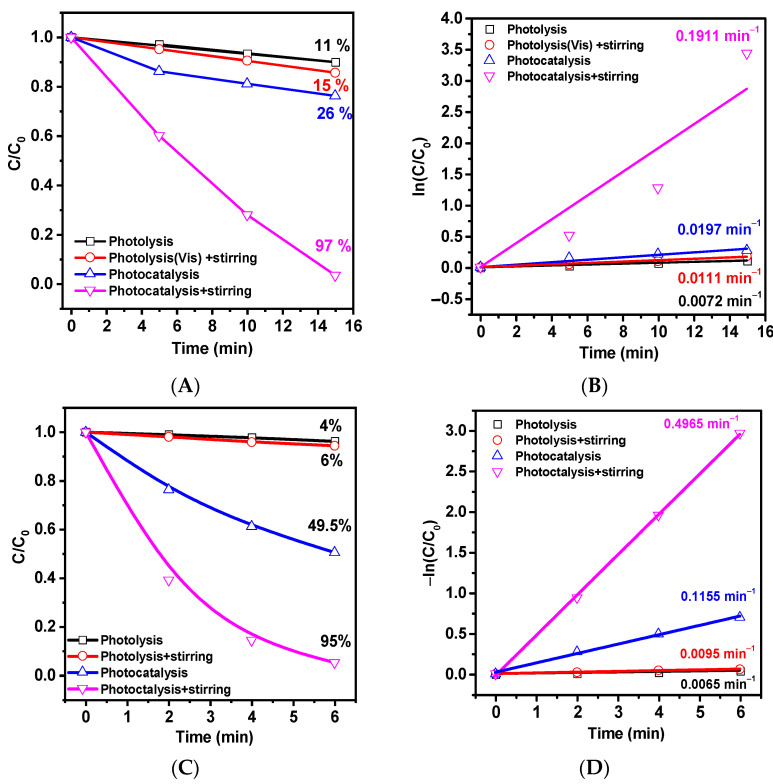
Changes in the concentration of MB and kinetic curves during irradiation with a metal halide lamp without cutoff filters for hydrophobic (**A**,**B**) and hydrophilic (**C**,**D**) ZnO-Ts.

The results of the photocatalytic decomposition of methylene blue (MB) using tetrapods in hydrophobic and hydrophilic states and metal halide lamp are presented in Figure 4.

As can be seen, even in a non-dispersed state without stirring, the ZnO-T shows photocatalytic activity, leading to a 26% degradation of MB in 15 min compared to 11% degradation under similar conditions during photolysis. The slight improvement in photolysis activity with stirring (15%) indicates the contribution of mass transfer processes. The photocatalysis with stirring showed that 97% of the dye decomposed in 15 min. The reaction was shown to accelerate by 10 times compared to photocatalysis without stirring (Figure 4B). The results of similar experiments on pre-hydrophilized ZnO-Ts are presented in Figure 4C. The highest activity was observed in the photocatalysis with stirring experiment, in which 95% of the MB was decomposed in 6 min. Without stirring, the catalysis efficiency dropped by almost half to 49.5%, while the degradation efficiency during photolysis was about 4–6%. The rate constants (k), calculated from the kinetic curves in Figure 4D using the pseudo-first-order equation, were 0.0065, 0.0095, 0.1155, and 0.4965 for photolysis, photolysis with stirring, photocatalysis, and photocatalysis with stirring, respectively. In photocatalysis (stirring), the reaction rate increased by 4.3 and 52 times compared to photocatalysis and photolysis, respectively. The significant acceleration of MB degradation on the hydrophilic ZnO-Ts (6.2 times for photocatalysis and 2.6 times for photocatalysis with stirring) compared to hydrophobic ones is due to both an increase in surface defects on the tetrapods and improved wettability, as all photochemical processes occur at the solid/liquid interface.

Considering the negligible amount of UV radiation present in the spectrum of the metal halide lamp, we conducted independent research utilizing cutoff filters to differentiate the impacts of both visible and UV light.

**Figure 5:**
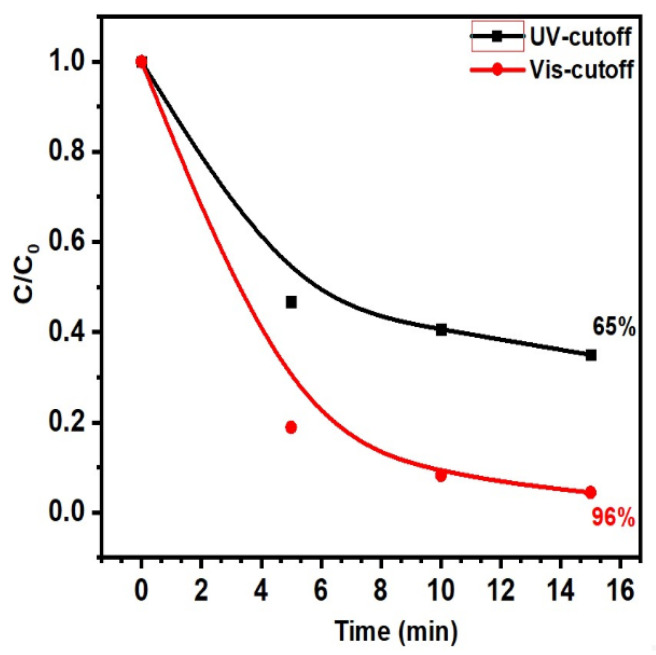
Changes in the concentration of MB via PC with UV- and Vis-cutoff filters for hydrophilic ZnO-Ts.

Figure 5 highlights the outcomes of our study, which reveal that exposure to UV light alone results in 96% degradation of MB within a 15 min timeframe. Conversely, cutting off UV light from the source leads to a decline in PC activity, with decomposition levels reduced to 65%. As a result, our findings suggest that ZnO-Ts in both visible and UV light possess PC activity. Photocatalytic activity in visible light can be influenced by surface oxygen vacancies and the photosensitization effect. Hydrophilization confirms the enhancing effect of oxygen vacancies on PC activity, while further experimentation is needed to verify the photosensitization effect.

**Figure 6:**
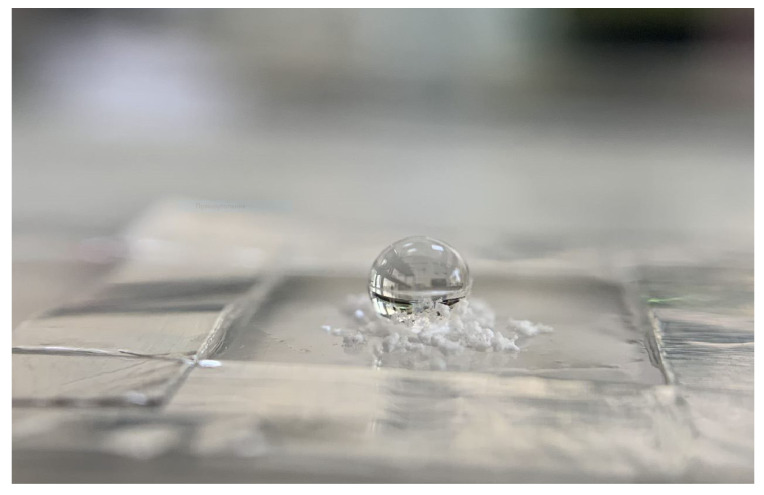
Photograph of a water droplet on the surface of hydrophobic ZnO-T powder.

Tetrapods, due to their unique morphology, are superhydrophobic and did not wet even during intense stirring of the solution in the experiment. For clarity, please refer to Figure 6, which shows a photograph of a water droplet on tetrapod powder on glass.

To understand the difference in the mechanisms of the photocatalytic reaction for hydrophilic and hydrophobic ZnO-Ts, tests were conducted to capture some active redox forms.

**Figure 7:**
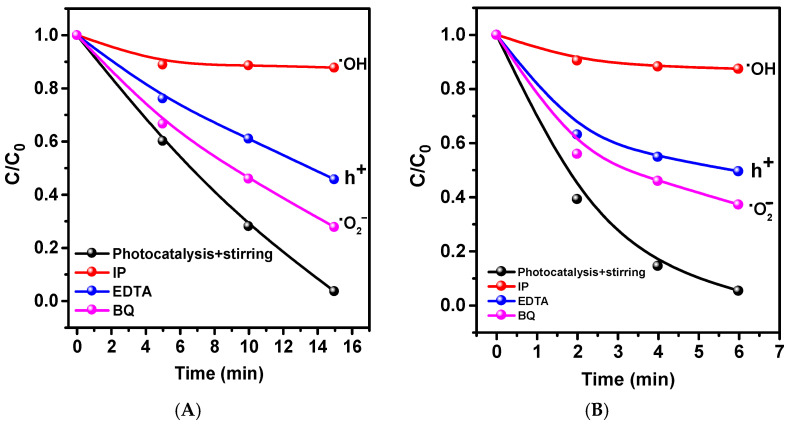
Influence of traps on changes in MB concentration during the photocatalysis process for hydrophobic (**A**) and hydrophilic (**B**) ZnO-Ts without cutoff filters, using a metal halide lamp.

From Figure 7, we can observe that hydroxyl radicals, which are produced in the hole–water reaction, significantly contribute to the decomposition mechanism. The holes themselves have a minor role since hydroxyl radicals are also generated through the reactions of superoxide anion radicals with water. The presence of AgNO_3_, an electron scavenger, enhances catalytic activity. This is because Ag accepts an electron and reduces silver, which can act as a co-catalyst in dye degradation. It is well established that Ag-ZnO composites catalyze the degradation of MB. The application of benzoquinone as a superoxide anion radical scavenger also results in a decrease in activity, indicating their generation and involvement in reactions.

**Figure 8:**
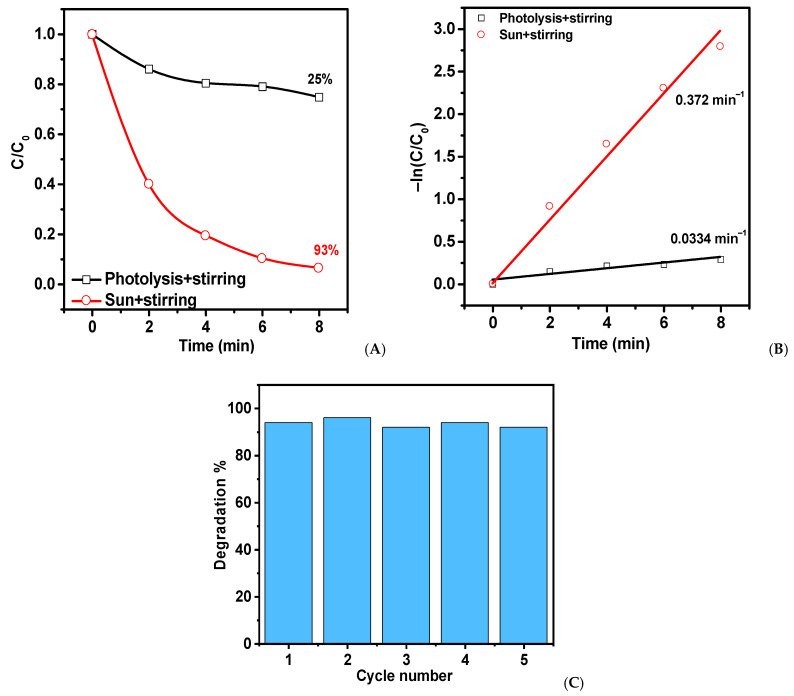
Changes in the concentration of MB (**A**) and kinetic curves (B) during solar photocatalysis on hydrophilic ZnO-Ts and a cyclic experiment (**C**).

A hydrophilic ZnO-T was tested for its photocatalytic activity with the stirring decomposition of MB under direct sunlight irradiation. Figure 8 presents the results, which show that in just 8 min, 93% of MB decomposed compared to only 25% during photolysis. Furthermore, the activity of the catalyst under sunlight is almost identical to that seen under lamp irradiation, with only a slight slowdown reaction of 1.3 times. Long-term stability tests were carried out under sunlight conditions. Figure 8C demonstrates that the photocatalyst’s activity minimally decreases, indicating exceptional stability.

Additionally, the PC degradation efficiency of these systems is evidently higher compared to previous studies, as shown in Table 1. Considering the studies exploring additional piezo stimulation in PC due to the piezo properties of ZnO, we present a comparative table comparing our PC results with piezophotocatalysis. Table 2 summarizes the findings.

The tables present a comparative analysis indicating the high photocatalytic activity of microtetrapods. Table 1 shows that comparable rate constants in photocatalytic reactions are achieved solely via UV radiation and ZnO modification. It is worth noting, though, that most materials presented in the studies were at a nanoscale, unlike the particles employed in our research, which have dimensions in the micron range. Compared with piezophotocatalysis that utilizes both UV radiation and ultrasonic treatment, as shown in Table 2, our materials exhibit high efficiency in terms of the rate constant.

**Figure 9:**
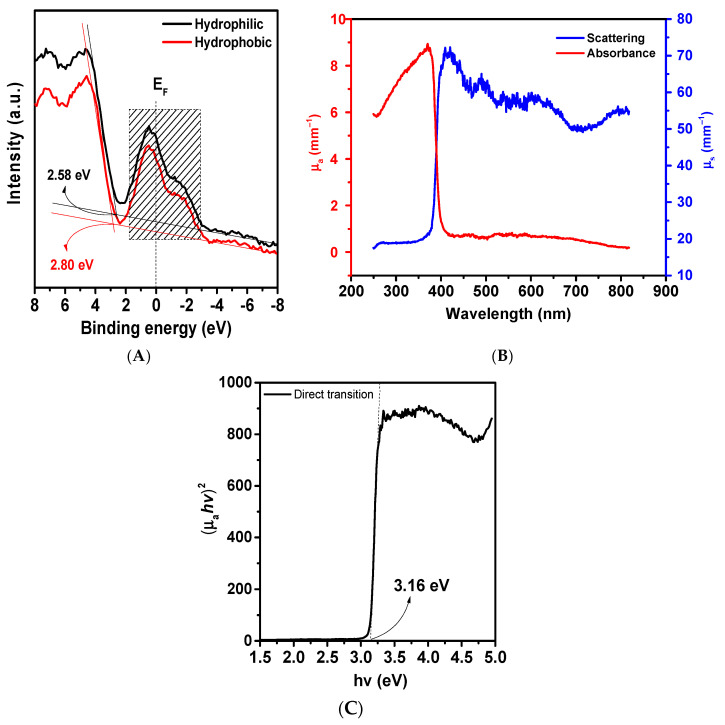
(**A**). VB XPS spectra for hydrophobic and hydrophilic ZnO-Ts. (**B**). Optical absorbance and scattering spectra. (**C**). Tauc plots for bandgap calculations.

It is well known that zinc oxide is a wide-bandgap semiconductor that cannot be excited by visible light. Optical investigations presented in Figure 9B,C reveal that the bandgap’s width is 3.16 eV. Furthermore, the material exhibits strong light scattering which surpasses the absorption coefficient throughout the entire wavelength range by an order of magnitude.

To determine the structure of energy zones, VB XPS spectra were obtained, and the data are presented in Figure 9A. The presence of energy state density localized near the Fermi energy is immediately noticeable, confirming the presence of oxygen defect levels in the bandgap. The VB_max_ energy was estimated to be 2.80 and 2.58 eV for the hydrophobic and hydrophilic states, respectively. Based on this, the CB_min_ was estimated to be −0.36 and −0.58 eV. The energy required to form surface layer defects is less than that of bulk defects, leading to a diffuse energy band linked to defects within the bandgap.

When light is applied, electron–hole charge states are generated due to localized levels of impurity in the bandgap, mainly caused by oxygen vacancies (V_O_). Electrons generated by the light and moving from the conduction band to the surface of the material will interact with dissolved oxygen in water, creating superoxide anion radicals which can efficiently oxidize organic pollutants or produce hydroxyl radicals. Upon exposure to light, photogenerated holes on the surface can either react directly with MB in water or produce hydroxyl radicals.

Consequently, the reaction mechanism can be expressed as follows, based on experimental data, and free charge carriers (e^−^/h^+^) are created:

(1) $$ZnO+hv+\to e^{-}+h^{+}$$

The existence of defect levels in the bandgap facilitates the primary capture of carriers and restricts recombination processes. Zinc defects within ZnO serve as electron traps for photoexcited electrons. These electrons can relax through interactions with oxygen molecules on the surface (reaction 3) or recombination. When electrons move toward the ZnO surface and interact with molecular compounds, superoxide radicals ·O_2_^–^ are formed (reaction 4). In the instance of hydrophilic ZnO-Ts, the involvement of adsorbed molecular oxygen also plays a part in this mechanism.

(2) $$e^{-}+O_{2}\to \cdot O_{2}^{-}$$

Similarly, holes (h^+^) are captured by oxygen vacancies and either interact with OH^–^/H_2_O on the surface to form ·OH (reactions 5, 6) or recombine.

(3) $$h^{+}+{OH}^{-}\to \cdot OH$$

(4) $$h^{+}+H_{2}O\to H^{+}+\cdot OH$$

These radicals further oxidize organic pollutants. Thus, the presence of photocatalytic activity under visible light indicates the role of defects, primarily oxygen vacancies, and a significant increase in photocatalytic activity with the addition of mechanical stress indicates the role of surface wettability, which is also influenced by increased surface defects.

## 3. Materials and Methods

### 3.1. Synthesis of ZnO Tetrapods

Crystalline powders of zinc oxide were prepared via a modified method of high-temperature pyrolytic synthesis. In the first stage, a filter impregnated with a ZnO precursor was rolled into tubes with a diameter of 5 mm and placed on mesh frames made of corundum rods in a porcelain container. Next, the containers were heated to 1150 °C in a muffle furnace with an air supply at a speed of 9 L/min. Heating was carried out with a temperature gradient of 3.7 °C/min. When the set temperature was reached, the heating was stopped and the system was kept in thermostatic mode for 30 min and then cooled to room temperature. An aqueous solution of zinc acetate Zn(CH_3_COO)_2_·2H_2_O (Alpha Aesar, Bio Aqua Group, Targu Mures, Romania) with a zinc concentration of 70 g/L was used as a ZnO precursor. An ash-free white tape filter with an ash mass of 0.15 wt.% (Himreactive, N. Novgorod, Russia) was used as a paper filter. The filter served as a source of carbon formation; carbon played the role of a reducing agent in the process of producing zinc vapor as an intermediate reaction product for subsequent oxidation and the formation of zinc oxide tetrapods. The resulting reaction products were removed from the frame.

### 3.2. Characterization of Samples

The surface morphology and chemical composition of the samples were investigated via scanning electron microscopy (SEM), using an FEI Quanta 200 3D microscope with an attached energy-dispersive X-ray spectrometer (EDS) and EDAX Genesis (accelerating voltage 20 kV). To prevent sample charging, the samples were fixed on the microscope stage using a conductive adhesive tape based on graphite.

Sample preparation for transmission electron microscopy (TEM) was performed on a “Scios” scanning electron ion microscope (SEM) (FEI, Lincoln, NE, USA). According to the standard methodology, cross-sections perpendicular to the central growth axis of the ZnO tetrapod rod-like protrusions were prepared. To protect the sample surface during preparation, a technological layer of Pt was applied on all sides of the sample at a thickness of 1–3 μm. The cross-sections were examined using an “Osiris” TEM (FEI, Lincoln, NE, USA) at an accelerating voltage of 200 kV in TEM mode, high-resolution electron microscopy (HRTEM) mode, and scanning transmission electron microscopy (STEM) mode, as well as an energy-dispersive X-ray spectrometer (EDS).

To determine the chemical composition of the zinc oxide and zinc oxide–titanium oxide composite via X-ray photoelectron spectroscopy (XPS), a SPECS XPS spectrometer (Specs, Berlin, Germany) equipped with an Al anode was used. The choice of anode material was made to avoid interference from Auger lines in the useful signal. Spectra were recorded in the binding energy range from 0 to 1200 eV. The calibration of binding energies was performed using the C-C line of the C1s spectrum (E binding = 284.6 eV).

X-ray diffraction patterns were obtained using a Rigaku Miniflex 600 diffractometer (Japan) with Cu-Kα radiation and a β-filter. The diffraction patterns were analyzed using the TOPAS software (Bruker, 2015).

The bandgap parameters were determined using UV/Vis spectroscopy, with a spectrometric complex based on the monochromator. The material powder was placed on a special holder and compacted. Diffuse reflection spectra were recorded in the wavelength range λ from 250 to 800 nm.

Cathodoluminescence (CL) excitation measurements were performed using an electron beam from an EG-75 electronograph, with an electron energy of 40 keV (spot diameter: 1 mm) and an electron beam current of 80 µA. The spectra were analyzed using the AvaSpec-ULS2048x64-USB2 spectrophotometric complex (Avantes, Apeldoorn, The Netherlands). A vacuum optical fiber coupler, an FC-VFT-UV400, was used to extract radiation from the electron column. The angle of incidence of the electron beam on the substrate plane was 45°, and the angle between the axis of the optical fiber coupler and the direction of propagation of the incident electron beam was 90°.

The EPR/ESR spectra of the studied samples were obtained using a Bruker EMX Plus radio spectrometer in the “X” ultra-high-frequency radio wave range (frequency, ~9.8 GHz; wavelength, ~3 cm) at room temperature. For all samples, spectra were recorded over a wide range of magnetic fields (0–6000 G) to examine the presence of all possible signals.

The spectra of the total transmittance T_t_ and diffuse reflectance R_d_ for the studied objects were measured in the wavelength range of (300 to 1000) nm using an Avasphere-50 integrating sphere (Avantes, Apeldoorn, the Netherlands). A combined deuterium/halogen lamp AvaLight-DH-S-BAL (Avantes, Apeldoorn, The Netherlands) was used as an illumination source; its radiation was supplied via 600 μm fiber-optic light guides. Photographic signals were registered using an automated spectrometer, an MS3504i (SOL-Instruments, Minsk, Belarus), coupled with a CCD matrix camera, an HS-101H-HR (Hamamatsu, Hamamatsu City, Japan). The final spectrophotometric coefficient data T_t_ and R_d_ were determined as follows:

(5) $$R_{d}^{exp}=\frac{R_{d}^{s}\left(\lambda \right)-R_{0}\left(\lambda \right)}{R_{gl}\left(\lambda \right)-R_{0}\left(\lambda \right)}$$

(6) $$T_{t}^{exp}=\frac{T_{t}^{s}\left(\lambda \right)-T_{0}\left(\lambda \right)}{T_{gl}\left(\lambda \right)-T_{0}\left(\lambda \right)},$$

where $T_{t}^{s}\left(\lambda \right)$ and $R_{d}^{s}\left(\lambda \right)$—are the transmission and reflection spectra of the samples; $T_{gl}\left(\lambda \right)$ and $R_{gl}\left(\lambda \right)$—are the spectra of the reference signal measured with quartz plates; $T_{0}\left(\lambda \right)$—is the signal of the integrating sphere with closed input and open output ports; and $R_{0}\left(\lambda \right)$—is the signal for the sphere with open optical ports. The spectral dependence of the optical absorption coefficient μ_a_ and light scattering coefficient—$\mu_{s}^{\prime }$ was calculated using an inverse Monte Carlo numerical modeling method, using the two-flow Kubelka–Munk model.

### 3.3. Photocatalytic Degradation Analysis

The photocatalytic characteristics of the samples were evaluated based on the photodegradation of methylene blue (MB) in an aqueous solution (2.5 mg L^−1^). Photocatalytic experiments were conducted in a 50 mL glass beaker. Visible and solar light was used in this case. A 70-watt metal halide lamp (Osram, Munich, Germany) was used as the light source. Activity with and without light filters that cut off wavelengths above and below 400 nm (λ > 400 nm and λ < 400 nm) was examined separately. A constant temperature in the reaction vessel of 26 °C was maintained using ventilation and monitored using a thermometer. For the photocatalytic reaction on hydrophobic particles, 20 mg of the original photocatalyst was added to 20 mL of an aqueous MB solution. Before turning on the light, the cuvette was kept in darkness for 60 min to achieve adsorption–desorption equilibrium. The photocatalysis process was carried out both without stirring and with stirring on a magnetic stirrer (400 rpm). The light source was positioned above the reactor at a distance of 10 cm. Samples (3 mL) were collected at fixed time intervals for each experiment. The particles were separated from the solution by centrifugation at 14,000 rpm for 2 min, using a laboratory centrifuge. The concentration of MB was measured using a spectrophotometer based on the characteristic absorption peak of MB at a wavelength of 663.7 nm. After the measurement, the solution was poured back into the reactor and the process was continued. For comparison, an MB solution was tested under similar conditions without a photocatalyst (photolysis). The concentration of MB was determined using the Beer–Lambert law.

For the photocatalytic reaction on hydrophilic particles, 20 mg of the original photocatalyst was initially poured into a beaker with distilled water (3 mL) and irradiated with a 250-watt high-pressure mercury UV lamp (Philips, Amsterdam, The Netherlands) without any cutoff filters until the complete evaporation of the water and the drying of the powder. The remaining experiment was conducted similarly to the hydrophobic one. Similar experiments were conducted under direct sunlight.

## 4. Conclusions

ZnO-Ts with surface oxygen defects were found to display exceptional photocatalytic activity in UV light, visible light, and direct sunlight. Controlling surface wettability was shown to regulate this activity, with an increase in surface defects occurring during the transition from hydrophobic to hydrophilic states. Although the optical width of the bandgap was 3.16 eV, the presence of a high density of localized defects in the bandgap led to sufficiently high PC activity in visible light. The experiment exhibited that using hydrophilic powder increases the reaction rate by 2.6 times compared to hydrophobic powder when irradiated with simulated sunlight. Implementing the procedure under direct sunlight results in a negligible reduction in the reaction rate by a factor of 1.3. Activity contributions from UV and visible light were distinguished by using cut-off light filters. In the presence of visible light, 65% of MB decomposes, while under UV light, 96% decomposes in 15 min. ·OH and ·O_2_^–^ radicals are the main active forms responsible for the degradation process.

## Figures and Tables

**Table 1:** Previously reported work and its comparison with our present work in the field photocatalytic properties of ZnO-based and other materials.

Materials	Pollutants	Time (min)	Light Source	Degradation, %	Rate Constant (min^−1^)	References
ZnO tetrapod1 ZnO tetrapod2	methyl orange methylene blue methyl orange methylene blue	130	UV lamp 100 W λ = 254 nm	50.8 85.7 61.6 96.4	1.6 × 10^−4^ 2.9 × 10^−4^ 1.7 × 10^−4^ 3.6 × 10^−4^	[28]
GNs-ZnO 20 mg	MB (1 mg/L)	40	UV (125 W) Vis (125 W)	70.87 17.26	4.5 × 10^−2^ 5 × 10^−3^	[29]
ZnO tetrapods 1 mg	MB (10 ppm)	70	UV light (Philips, 350–400 nm wavelength, 60 W)	94.5	1.02 × 10^−1^	[30]
ZTPG	Methylene blue (20 ppm)	90	UV light (60 W, 365 nm)	98.05	0.03	[31]
ZnO sample 25 mg	V = 100 mL Rhodamine B (20 ppm)	110	UV irradiation (8 W)	98.86	0.036	[32]
T-ZnOw/PLLA	V = 50 mL MB (3 × 10^−4^ M)	60	Visible light	30	0.0065	[33]
T-ZnO 50 mg	V = 50 mL MB (5 mg/L)	8	UV illumination (365 nm, 66.2 mW/cm^2^, Blak-Ray B-100 AP lamp)	100	-	[34]
Rod-like ZnO nanoparticles 10 mg	V = 50 mL MB (50 mg/L)	120	100 W halogen lamp (with λ > 420 nm and a light intensity of 2.87 W m^–2^)	30.67	2.9 × 10^−3^	[35]
T-ZnO T-ZnO-CNO 100 mg	V = 50 mL DNP (0.1 mM)	140	60 W tungsten bulb	30 92	0.00274 0.01834	[36]
ZnO tetrapods 60 mg	V = 60 mL MB (1 μmol·L^–1^)	10	UV diode array consisting of four diodes (central wavelength = 370 nm, 170 mW/diode)	96	-	[37]
NWs TNFs coated Si substrates of area 0.5 cm^2^	V = 10 mL RhB (5 × 10^–6^ M)	180	100 W bulb (with a luminous irradiance of 10 mW/cm^2^ at the sample) λ ≥ 400 nm	95	-	[38]
T-ZnO Ag_2_O/T-ZnO 200 mg	V = 100 mL MB (5 mg L^−1^)	2	UV lamp, 50 W	63 85	- -	[39]
ZnO_1−x_ 50 mg	V = 100 mL MB (1 × 10^−5^ M)	360	Halogen–tungsten lamp (power = 175 W; λ_main_ = 550 nm	95	0.522 h^−1^	[40]
MoS_2_/Ag-ZnFe_2_O_4_ 40 mg	V = 50 mL TC = 10 mg/L	60	300 W Xenon lamp with optical filter (λ ≥ 420 nm)	95	0.04868	[41]
2D g-C_3_N_4_ nanosheets 20 mg	V = 100mL TEOA = 10%	240	300 W Xenon lamp with optical filter (λ ≥ 420 nm)	-	7.414 mmol g^−1^ h^−1^	[42]
SrTiO_3_ 50 mg	120 mL of 25% aqueous methanol solution	300	UV–visible light 300 W Xenon lamp	-	2.2 mmol h^–1^ g^–1^	[43]
BiPO_4–x_ 25 mg	V = 50 mL MB = 1 × 10^–5^ M	30	UV-light 300 W high-pressure mercury lamp	89	0.300	[44]
Bi_2_MoO_6_ 20 mg	V = 50 mL CIP = 20 ppm	40	300 W Xenon lamp with optical filter (λ ≥ 400 nm)	97	1.7990 mg m^−2^ min^−1^	[45]
TiO_2-x_/Ag_3_PO_4_ 100 mg	V = 100 mL BPA = 10 mg/L	16	500 W Xenon lamp with optical filter (λ ≥ 420 nm)	95	-	[46]
			70W metal–halogen lamp:			
ZnO-T 20 mg	20 mL of MB (2.5 mg/L)	6 15 15 8	Without cut-off λ > 400 nm λ < 400 nm Direct sunlight	95 65 96 93	0.496 0.101 0.229 0.372	This work

**Table 2:** Previously reported work and its comparison with our present work in field of the piezophotocatalytic properties of ZnO-based and other materials.

Materials	Pollutants	Time (min)	Light/Mechanical Source	Degradation, %	Rate Constant, (min^−1^)	References
ZnO NS/ 2.5 mg	V = 10 mL TST (testosterone) (5 × 10^−5^ M)	45	LOT-Oriel Solar S (140 W), 35 kHz	50	1.8 × 10^−2^	[47]
ZnO nanowires/CFs 200 mg	V = 100 mL MB (C_0_ = 5 mg/L)	120	High-pressure mercury lamp (50 W)/stirring	96	-	[48]
ZnO nanorods 20 mg	V = 50 mL RhB (10 ppm)	20	300 W Xe lamp equipped with a 350 nm bandpass filter/ultrasonic frequency 27 kHz	75	0.0744	[49]
calcined ZnO_TW-0.20_ 1g/L	V = 100 mL MB (5 ppm)	120	UVA light with peak wavelength of 365 nm and intensity of 940 μW cm^−2^/ultrasonic bath (120 W, 40 kHz)	90	-	[50]
T-ZnO nanostructures 200 mg	V = 100 mL MB (5 mg L^−1^)	2	UV lamp/ultrasonic probe 50 W UV, 200 W ultrasonic	74	-	[51]
Bi_2_VO_5.5_ 0.25 g	V = 10 mL MB = (5 mg/L)	240	15W (Havells company) 2 lamp visible light; ultrasonicator (40 kHz, 150 W).	82	0.00528	[52]
FTO/BaTiO_3_ /AgNPs 2 cm × 2 cm	V = 75 mL MB = (5 mg/L)	180	70 W UV lamp; 24 kHz ultrasonic vibration 30 W	90	0.02329	[53]
BaTiO_3_ –NiO 0.2 g	V = 200 mL MB = (10 mg/L)	80	UV lamp, 125 W; ultrasonic cleaner, ~40 kHz	90	0.028	[54]
ZnO/ZnS/MoS_2_ 10 mg	V = 50 mL MB = 10 mg/L	50	300 W Xenon lamp to simulate the solar source; stirring at 1000 rpm	87	0.0411	[55]
BiVO_4_ 0.2 g	V = 10 mL MB = (5 mg/L)	240	15W (Havells company) 2 lamp visible light; ultrasonicator (40 kHz, 70 W).	81	0.00802	[56]
CuS/ZnO nanowires on stainless steel mesh 6.0 × 6.0 cm, 100 mg	V = 50 mL MB = 5 mg/L	20	Xenon lamp, 500 W, to simulate the solar source; ultrasonic probe, 200 W	98	0.18236	[57]
BaTi_2_O_5_ 40 mg	V = 60 mL RhB = 10 mg/L MB = 10 mg/L MO = 10 mg/L	50	Xenon lamp, 300 W, λ > 400 nm; ultrasonic cleaner, 53 kHz, 100 W	82.5 - -	0.0353 0.1775 0.0314	[58]
ZnO/ZnS	V = 50 mL MB = 5 mg/L	50	300 W UV irradiation; 180 W sonication, 40 kHz	60	0.0154	[59]
			70 W metal–halogen lamp:			
ZnO-T 20 mg	V = 20 mL MB (2.5 mg/L)	6 15 15 8	Without cut-off λ > 400 nm λ < 400 nm Direct sunlight	95 65 96 93	0.496 0.101 0.229 0.372	This work

## Data Availability

The data are available upon request.
